# Supplementary material for: Edaravone-Loaded Alginate-Based Nanocomposite Hydrogel Accelerated Chronic Wound Healing in Diabetic Mice
Source: Mar Drugs. 2019 May 11;17(5):285. doi: 10.3390/md17050285 (PMC6562986; doi:10.3390/md17050285)
Supplement: Supplementary file 1 [file marinedrugs-17-00285-s001.pdf]

marine drugs

Article

Edaravone-loaded alginate-based nanocomposite hydrogel accelerated chronic wound healing in diabetic mice

Ying FAN[1], Wen WU[1*], Yu LEI[3], Caroline GAUCHER[2], Shuchen PEI[3], Jinqiang ZHANG[1], Xuefeng XIA[1*]

[1] Chongqing Key Laboratory of Natural Product Synthesis and Drug Research, School of Pharmaceutical Sciences, Chongqing University, Chongqing 401331, China; fanying@cqu.edu.cn (Y.F.); j.zhang1983@cqu.edu.cn (J.Z.)
[2] School of Chemistry and Chemical Engineering, Chongqing University of Science and Technology, Chongqiang 401331, China; backer7_leiyu@163.com (Y.L.); peishuchen928@163.com (S.P.)
[3] Faculté de pharmacé, Université de Lorraine, CITHEFOR F-54000 Nancy Cedex, France; caroline.gaucher@univ-lorraine.fr (C.G.)

* Correspondence: wuwen1988@cqu.edu.cn (W.W); xxia@cqu.edu.cn (X.X.)

Received: date; Accepted: date; Published: date

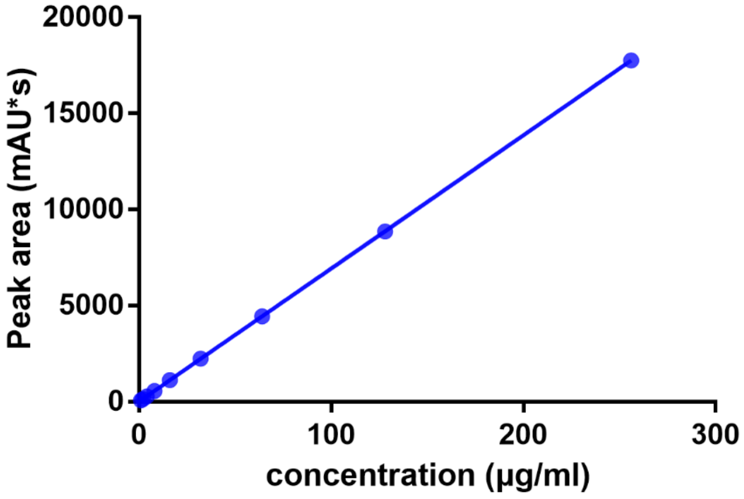

Figure S1. The standard curve of edaravone was established by High Performance Liquid Chromatography. (y = 69.332x + 5.3527, $R^2 = 1$).

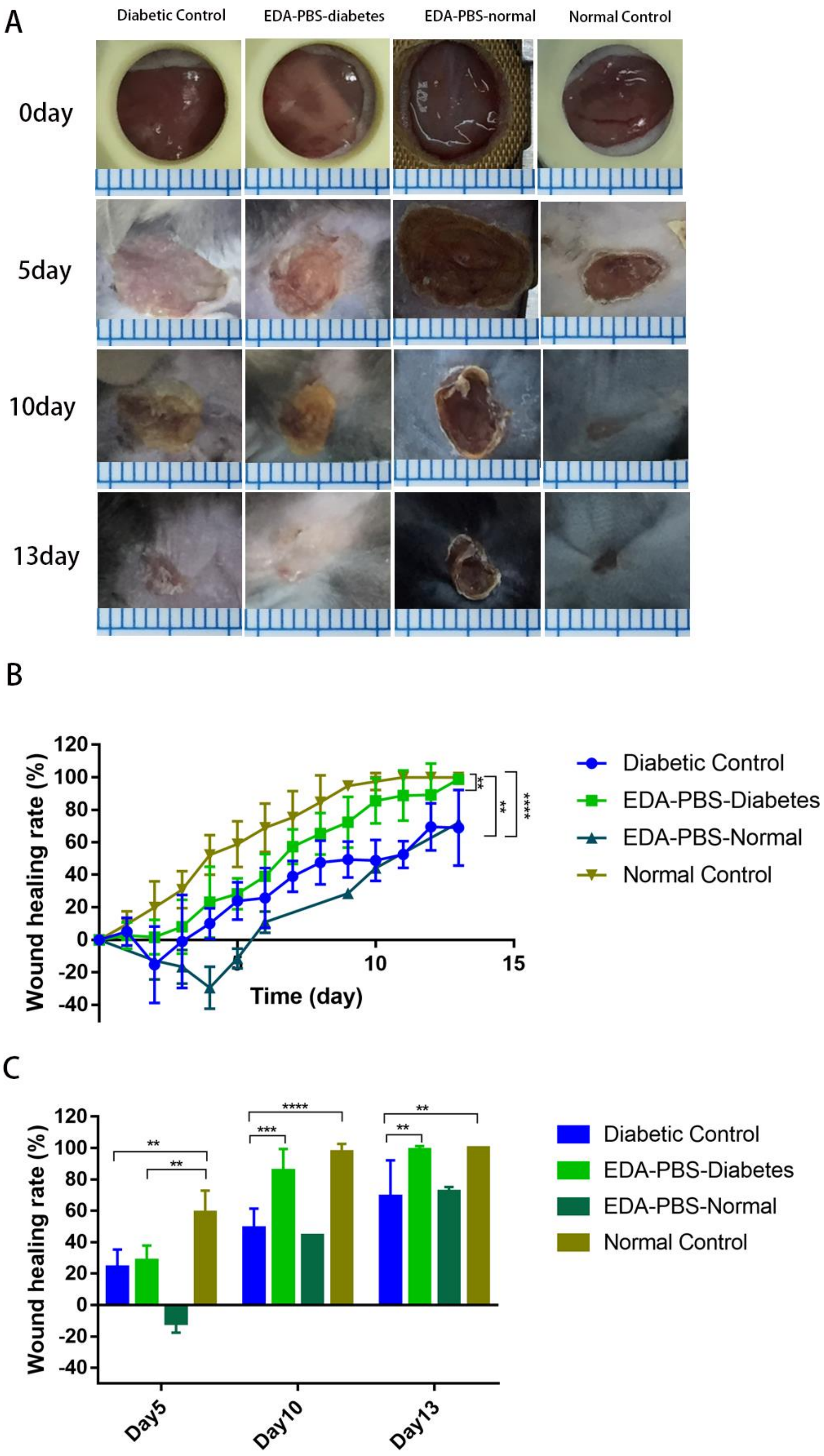

Figure S2. Effects of edaravone on wound closure in diabetic mice and normal mice. (A) Representative images of wounds of four groups. (B) Healing progression for day 0–13. (C) Percentage wound closure on day 5,10,13 after wounding. (mean ± SD, n=4). (**P < 0.01, ***P < 0.005; ****P < 0.001).

A

B

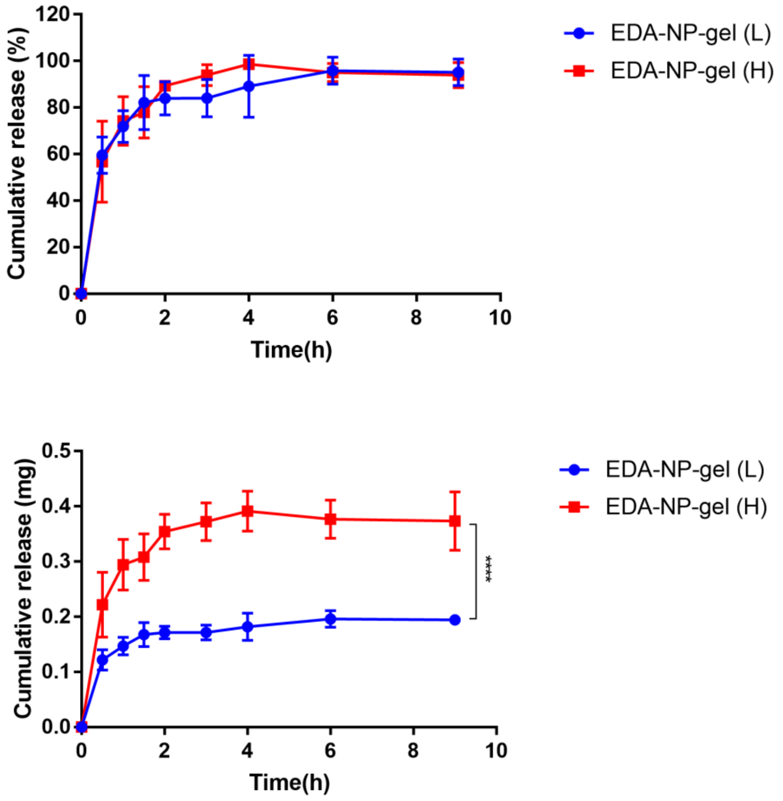

Figure S3: In vitro release behavior of high and low dose of edaravone loaded nanocomposite hydrogel using dialysis bag method in phosphate buffer (pH 5) at 37℃. A: the percentage of edaravone released. B: the absolute amout of edaravone released. The edaravone concentrations were measured by high performance liquid chromatography (mean ± SD, n=3, ****$p < 0.001$).
